# Hospital Saturday, 1915

**Published:** 1915-10-16

**Authors:** Charles Johnston

**Affiliations:** Lord Mayor. Mansion House, London, E.C.


*To the Editor of* The Hospital.

### Hospital Saturday, 1915.

*To the Editor of* The Hospital.

Sir,—In these times one is diffident in asking for help even for an association which has been established for nearly half a century. Knowing, however, the never-failing charity of the public, I make the annual appeal on behalf of the Hospital Saturday Fund. Since it was established it has contributed £672,571 to the medical charities of London. During 1914 it distributed among its subscribers and their dependants 52,334 benefits, including treatment at the hospitals, at sanatoria, and at convalescent homes; the provision of surgical appliances, first-aid requisites, and dental treatment. The total sum received in 1914 was £40,676. In all sections of its work the Fund inculcates the principles of self-help.

As an indication of the spirit of its weekly supporters, I may mention that its income so far this year amounts to £1,800 in excess of that for the corresponding period last year. The splendid manner in which the hospitals are succouring our sick and wounded soldiers has appealed to their patriotism; if the Fund has lost thousands of supporters through the war, it has gained other thousands at home who are engaged in munition and war work. I have the utmost confidence in appealing in support of the Metropolitan hospitals through the medium of the Hospital Saturday Fund.

Donations may be sent to the Mansion House, or to the Honorary Treasurer, the Hon. H. L. W. Lawson, M.P., 54 Gray's Inn Road, London, W.C. —I am, Sir, your obedient servant,

Charles Johnston, Lord Mayor.

Mansion House, London, E.C.

October 13, 1915.

